# On the duality between interaction responses and mutual positions in flocking and schooling

**DOI:** 10.1186/s40462-014-0022-5

**Published:** 2014-10-25

**Authors:** Andrea Perna, Guillaume Grégoire, Richard P Mann

**Affiliations:** Paris Interdisciplinary Energy Research Institute, Paris Diderot University, 10 rue Alice Domon et Léonie Duquet, Paris, 75013 France; Laboratoire Matiere Systemes Complexes, Paris Diderot University, 10 rue Alice Domon et Léonie Duquet, Paris, 75013 France; Mathematics Department, Uppsala University, Lägerhyddsvägen 1, Uppsala, 75754 Sweden; Chair of Sociology, in particular of Modeling and Simulations, ETH Zürich, Clausiusstrasse 50, Zürich, 8092 Switzerland

**Keywords:** Collective motion, Schooling, Flocking, Movement analysis

## Abstract

Recent research in animal behaviour has contributed to determine how alignment, turning responses, and changes of speed mediate flocking and schooling interactions in different animal species. Here, we propose a complementary approach to the analysis of flocking phenomena, based on the idea that animals occupy preferential, anysotropic positions with respect to their neighbours, and devote a large amount of their interaction responses to maintaining their mutual positions. We test our approach by deriving the apparent alignment and attraction responses from simulated trajectories of animals moving side by side, or one in front of the other. We show that the anisotropic positioning of individuals, in combination with noise, is sufficient to reproduce several aspects of the movement responses observed in real animal groups. This anisotropy at the level of interactions should be considered explicitly in future models of flocking and schooling. By making a distinction between interaction responses involved in maintaining a preferred flock configuration, and interaction responses directed at changing it, our work provides a frame to discriminate movement interactions that signal directional conflict from interactions underlying consensual group motion.

**Electronic supplementary material:**

The online version of this article (doi:10.1186/s40462-014-0022-5) contains supplementary material, which is available to authorized users.

## Background

Several animal species exhibit forms of collective motion in which two or more individuals move together coherently. Examples include flocks of migrating birds, schools of fish, murmurations of starlings, swarms of locusts, and many others. In general, the same group of animals can produce various types of collective patterns, including disordered aggregations, milling, or schooling depending on both internal states (e.g. hunger level) and external conditions (e.g. in response to a predator).

Much of our current understanding of collective motion of animal groups comes to us from the study of theoretical models, and in particular of a class of models known as ‘self-propelled particle models’. These models indicate that a small set of ‘rules’ of interaction is sufficient to generate group level patterns that resemble, at least visually, those formed by real animal groups. For instance, Reynolds [1] proposed a model that implements only three different rules. The first rule consists in a *repulsion* behaviour, through which each individual turns away from its local neighbours and avoids local crowding and collisions. The second rule is an *alignment* behaviour, or a turning response towards the average heading of local neighbours. The third rule is a turning response towards the position of more distant neighbours; this is an *attraction* rule, that contributes to maintain the members of the group together. Several alternative models of collective motion have been proposed (see [2] for a review), each implementing a slightly different set of interaction rules. In spite of their differences, almost all the models existing in the literature are able to produce realistic looking patterns of collective behaviour, at least within a certain range of parameters.

In order to make meaningful predictions about the collective movement patterns of a given animal species, it is important that the interaction rules implemented in the models match those actually used by animals of that particular species. In order to determine how real animals of different species interact together, several research groups have started to collect empirical data on the movement patterns of real animal groups. Traditionally, this has been done either focusing on the collective level, or on the individual level. The collective-level approach consists in collecting data on the spatio-temporal organization of the group, such as e.g. the mutual positions of close neighbours, and testing which theoretical models are compatible with the data; the individual-level approach operates instead by selecting a ‘focal individual’ within the group, and recording all the changes of speed and direction of movement of that individual in response to the position and movement of its neighbours [3]. Here, we provide a brief review of this literature, with particular emphasis on articles that either measure or predict the mutual positions of close neighbours.

As an example of the collective-level approach, Ballerini et al. [4] tracked the 3D positions of starlings flocking together in natural flocks, with the aim of characterising the spatial organization of the group. These authors observed that nearest neighbours consistently occupy the same positions with respect to each other, determining an anisotropic arrangement at the local scale. The anisotropy did not spread to the scale of the entire flock, but dropped quickly to a completely isotropic distribution between the sixth and the seventh nearest neighbour. The fact that the anisotropy cut-off depended on the number of neighbours, but not on the density of the group, was interpreted as evidence that starlings ‘pay attention’ to a fixed ‘topological’ number of six - seven neighbours, instead of responding to all neighbours within a fixed ‘metric’ distance. The topological nature of interactions in starlings was later confirmed also by an alternative maximum entropy approach, based on the relative alignments of nearest neighbours, instead of their positions [5]. A similar collective level approach was adopted by Lukeman et al. [6]. These authors recorded the positions and orientations of surf scoters sitting on the water surface. The observed arrangements of neighbours around a focal individual were consistent with models implementing repulsion, alignment, and attraction, but also required the existence of a more direct interaction with one single neighbour situated in front. Buhl et al. [7] measured the relative positions of swarming locusts, and observed isotropy in the radial distribution of neighbours around a focal individual. This distribution was compatible with both metric and topological models of interactions, but not with a third class of ‘pursuit/escape’ models [8] in which individuals try to reach neighbours ahead of and moving away from them, while they escape from other individuals that approach them from behind. Hemelrijk et al. [9] measured how the overall shape (length *vs.* width) of schools of mullets scales with group size. Their empirical data were consistent with a model in which the oblong shape of some schools, results from individuals slowing down to avoid collisions.

As examples of studies that have adopted the individual-level approach, we can mention Katz et al. [10], who reconstructed the ‘force maps’ that describe the acceleration and turning of schooling golden shiners, and Herbert-Read et al. [11], who reconstructed the force maps of mosquitofish. These studies indicated that a fundamental component of how fish of both species interact are changes of speed: the fish consistently increased or decreased their speed to catch neighbours that they had respectively in front or behind; but when a neighbour was too close by, the speed responses were reversed, so speed changes also mediated collision avoidance. Both studies found only weak alignment responses, in comparison to attraction and repulsion forces. While both mosquitofish and golden shiners formed aligned groups, this was more a consequence of the fish following each other (and eventually becoming aligned) than an explicit alignment response. More recently, Pettit et al. [12] applied a similar approach to the study of flight interactions in pigeons. The observed flocking responses of pigeons where different from those found in fish: alignment responses were explicit and strong, and collision avoidance was mainly mediated by turning, while speed remained relatively constant. These observations could be interpreted in terms of the different needs and constraints associated with flocking, which are different from those experienced by fish during schooling. Explicit alignment responses, for instance, might be necessary to achieve the high cohesion of pigeon flocks, that can fly without splitting for several kilometers. Avoiding collisions by turning away from the neighbour, instead of slowing down, might respond to a necessity to maintain a relatively constant speed, required to produce a sufficient lift force.

Gautrais et al. [13] used an intermediate approach to build a model of the shoaling behaviour of fish: they first characterized the motion of isolated fish, and progressively added interaction terms to the model through visual observations of how fish interact with obstacles and other fish, using quantitative methods to fit the parameters of these interaction rules to the tracked movements of the fish. The model was then tested at the collective level, by collecting statistics of the alignment and distance of real fish. In spite of its nice data driven formulation and good fit to experimental data, the model introduced by these authors does not formulate predictions about the mutual positions of nearest neighbours, and does not quantify these mutual positions in the empirical data; for these reasons it will not be discussed further in the context of our simulations which insist precisely on these aspects.

While some work has characterised directly the interaction responses of individuals, and other work has derived interaction responses indirectly, by selecting the interaction rules that reproduced better the observed configuration of a group, it is clear that different interaction rules lead naturally to different local configurations of the group. Consider for instance the case of an animal that avoids collisions by changing speed (like mosquitofish or golden shiners). Its acceleration response will be positive when the neighbour is in front and negative when the neighbour is behind, but will invert sign in the repulsion zone. The only region where there is no acceleration response is on the border between attraction and repulsion zone. Similarly, if turning does not mediate collision avoidance, the turning response will be simply directed towards the neighbour, that is, to the left if the neighbour is on the left and to the right if the neighbour is on the right. There are only two ‘fixed points’, for which both the turning and the acceleration response are zero: one directly in front and the other directly behind the neighbour. Not surprisingly, these positions are those at which both mosquitofish and golden shiners are most likely to have their neighbours [10,11]. Similar arguments can be used to explain that when collision avoidance is mediated through turning away from the neighbour (as in pigeons), a side by side configuration is the one which is stable (this is the configuration that was most frequently observed in pigeons [12]). In other words, different interaction rules lead naturally to different local arrangements of neighbours within the group.

In the present paper, we examine the different implications of this duality between interaction responses and mutual positions in flocks and schools. Unlike in previous studies, where mutual positions result from the interactions, here we consider the theoretical situation of animals maintaining stable mutual positions, and we address the question of what ‘apparent’ interaction responses would be observed as a mere consequence of the imposed mutual positions and noise.

## Introduction

**Figure 1:**
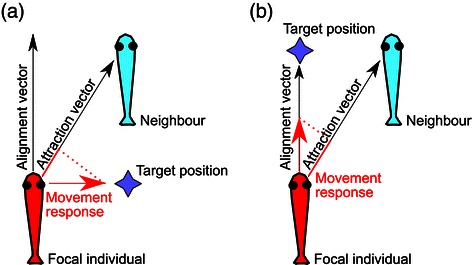
**Illustration of the interactions.** The focal fish (in red) aims at keeping a stable target position relative to its neighbour. In **(a)** this target position is behind the neighbour, while in **(b)** it is on the side of the neighbour. The movement in the direction of the target can be interpreted in terms of attraction or repulsion response if it has a projection onto the attraction/repulsion vector pointing in the direction of the neighbour. If the movement response has a component along the direction parallel to the neighbour (the alignment vector), it can also be interpreted as alignment. In general, the attraction/repulsion vector and the alignment vector are not orthogonal to each other, and in the particular case of aligned individuals with target positions in front or behind, the attraction and alignment vectors are not even linearly independent.

The movement of a focal individual with respect to a neighbour can be decomposed into an alignment response and an attraction-repulsion response by projecting it onto two different vectors (see Figure 1). Alignment is the component of movement response that has the same bearing as the neighbour. Attraction and repulsion correspond to the projection of focal individual’s movement on the vector oriented towards its neighbour’s body. In general, these two vectors are not orthogonal, except in very specific situations, such as when the focal individual and the neighbour move side by side in the same direction. In the extreme case when the focal individual and the neighbour are one behind the other, the alignment and the attraction/repulsion vectors coincide.

If the focal individual aims at keeping a fixed ‘target position’ relative to its neighbour, for instance on its side, or behind it, we can imagine that it will spend most of the time in the proximity of that position, repeatedly moving away from it under the effect of noise, and actively heading back to it. Movements away from the target position, or back to it, can correspond to real animal movements, but can also result from noise associated with recording the position of the focal individual, such as GPS inaccuracy (in case of GPS tracking), or segmentation variability and pixelization (in case of video tracking).

Figure 1-(a) shows a specific example with one individual, in red, having a preference for being directly behind its neighbour (target position marked by a star). A turn in the direction of the target position will be interpreted as an attraction (or repulsion) response; conversely, an alignment response would require to keep a straight direction, but this is not compatible with approaching the target. In Figure 1-(b), the relative positions of the focal individual and of its neighbour are the same, but the focal individual aims at reaching a schooling configuration side by side with its neighbour. The corresponding movement would be described in terms of an alignment response (the focal individual remains parallel to its neighbour), but also of attraction (because in this example reaching the target position involves getting closer to the neighbour). Both examples depict the same type of response (an attraction to the target), but we interpret them in terms of different alignment and attraction responses because we consider the other individual and not the target as the ‘point of attraction’.

**Figure 2:**
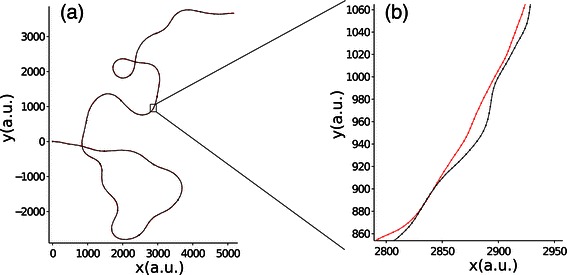
**Example of generated trajectories for two particles moving side by side.** **(a)** Complete trajectory of 2^12^ steps. The larger dots (visible when zooming in the figure) indicate the scale for temporal correlation *C*_*T*_ (= 300 steps) used for generating the trajectories. **(b)** Zoom on a smaller portion of trajectory to illustrate the recorded positions of both individuals. Each dot represents the position at one different time step.

The actual situation of two individuals moving together in two or three dimensions is more complicated, and involves not only different types of interactions e.g. alignment and/or attraction/repulsion, but also different types of responses, e.g. through turning, or acceleration, or both. In addition, in a real flocking situation individuals are not always aligned with each other and can have different speeds, making it more difficult to predict what interaction rules appear, on average, over a common trajectory. To test what interaction responses might support the movement of particles flocking together at a fixed distance and relative bearing, we simulate particles moving on the same trajectory but subject to small random displacements around these target positions (see Methods). In particular, we focus on two configurations: one in which the two particles fly side by side, and one where the two particles fly one behind the other. Figure 2 illustrates one such generated trajectory for two particles moving side by side.

## Results

**Figure 3:**
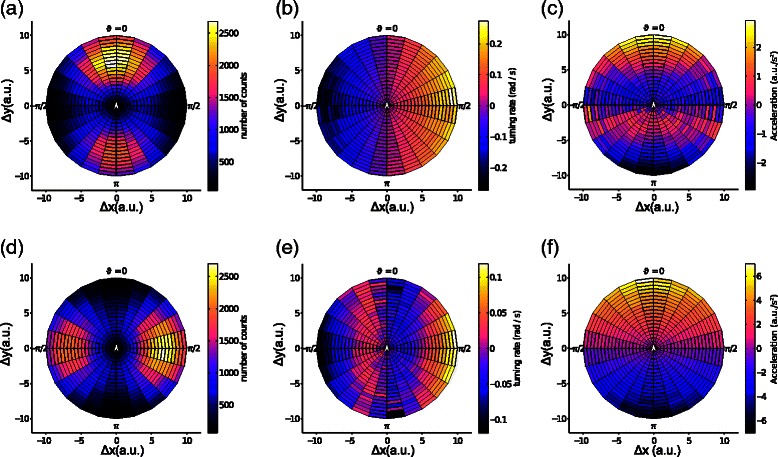
**Inferred interaction rules as a function of distance and direction to the neighbour.** In all these plots, the focal individual can be imagined to be situated in the centre of the plot, heading towards the top of the page, and the coordinates of each cell in the polar grid correspond to the position of the neighbour. **Top row** Individuals moving in a front-back configuration. **Bottom row** Individuals moving side by side. **(a)** and **(d)** Number of counts of the neighbour within each cell of the polar grid. The positions at which the neighbour is most frequently observed match those imposed when generating the trajectories. **(b)** Turning response. When the individuals move in a front-back configuration, turning always happens in the direction of the neighbour. **(c)** Acceleration response for individuals moving in a front-back configuration. Close-by neighbours elicit a repulsive response, with an acceleration of the opposite sign. **(e)** Turning response of individuals moving side by side. Repulsion is mediated through turning away from the neighbour. **(f)** Acceleration response. For individuals moving side by side, acceleration is always positive when the neighbour is in front and negative when the neighbour is behind.

As expected, when the trajectories are arranged in a side-by-side or in a front-back configuration, this same configuration is observed in the positions at which the neighbour is frequently observed (Figure 3-(a) and (d)). When the two trajectories are arranged in a front back configuration, the focal individual appears to turn in the direction of its neighbour with no ‘repulsion zone’: independently of distance there is no zone in which turnings are directed away from the neighbour (Figure 3-(b)). In this case, repulsion is mediated instead by changes of speed, as it is visible in Figure 3-(c), where acceleration is positive for neighbours situated in front and negative for neighbours situated behind, but there is a region in which the polarity of the acceleration response is inverted, when the front-back distance to the neighbour is smaller than 5 arbitrary units (the target distance between neighbouring particles implemented in the trajectories). These patterns of response are inverted for side by side trajectories: in this case, collision avoidance appears to be mediated through turning (Figure 3-(e)), while changes of speed mediate attraction, but not collision avoidance (Figure 3-(f)).

Our plots are similar to those obtained for real animal species, e.g. by Katz et al. [10] and Herbert-Read et al. [11] for fish moving prevalently in a front-back configuration and by Pettit et al. [12] for pigeons flying side by side. The main difference is that in all studies on real animals, the repulsion zone had a roughly circular form, centered around the focal individual, while in our plots the repulsion zone has the form of a band, parallel or perpendicular to the direction of movement of the focal individual. This difference is likely due to the fact that in our simulations, the underlying trajectories of the two particles are never exchanged for the entire duration of one “flight”: one individual has its attractor always on the left side of its partner and the other individual always on the right side (or one individual always in front and the other always behind). Real animals do switch from one to the other side of their neighbour (or from being in front to being behind), which means for instance that an animal situated roughly behind its neighbour (*𝜗*≃0 in Figure 3-(e)), and aiming at being on its side, will be nearly equally likely to turn left as to turn right, and on average will exhibit no consistent turning response.

**Figure 4:**
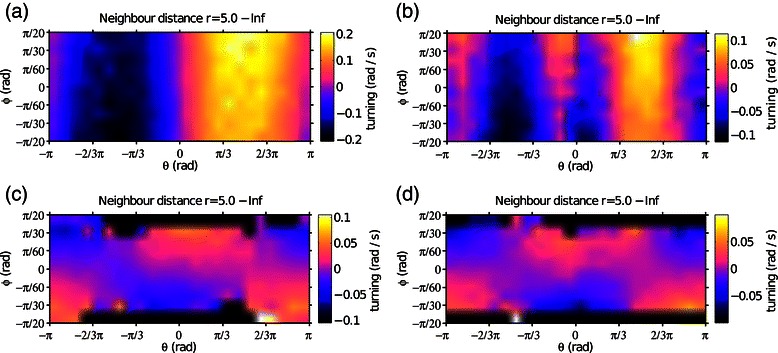
**Relative effect of ‘attraction’ and ‘alignment’.** The figures represent the average turning angle of the focal individual in response to the direction (*θ*) and relative bearing (*ϕ*) of the neighbour, limited to situations in which the neighbour is in the attraction zone (at a distance *r*>5 a.u.). Values of *θ* close to zero indicate that the neighbour is in front of the focal individual, with positive values indicating that the neighbour is on the right and negative values indicating that the neighbour is on the left of the focal individual. Positive values along the alignment axis *ϕ* indicate that the neighbour is oriented to the right, with respect to the focal individual, while the two individuals are aligned for values of *ϕ* close to zero. **(a)** Condition in which the two particles fly in a front-back configuration. **(b)** Particles flying side by side; **(c)** Same condition as **(a)**, but with increased temporal autocorrelation of noise around the target position (*C*_*D*_=100 steps, while it was *C*_*D*_=20 steps in the previous plots). **(d)** Same as **(b)**, with increased temporal autocorrelation of noise.

Figure 4 plots the turning angle of the focal individual as a function of the direction of the neighbour (with respect to the moving direction of the focal individual) and relative bearing (difference of alignment). The figure is limited to the data points for which the focal individual has its neighbour in the attraction zone, i.e. when the mutual distance between the two individuals is larger than the average distance implemented in the trajectories (The Matlab®; code that we provide as electronic Additional file 1 has an easy to run interface to plot responses to neighbours both in the attraction and in the repulsion zone, including acceleration responses and responses of individuals having different target positions).

When the trajectories are arranged in a front-back configuration (Figure 4-(a)), the focal individual shows a strong turning response to face its neighbor’s position, while alignment with the orientation of neighbors is not so much in evidence: the turning response in the figure is modulated along the attraction (*θ*) axis, but presents almost no modulation along the alignment (*ϕ*) axis. In the case of trajectories arranged side by side (Figure 4-(b)), the alignment response remains weak (modulation prevalently along the *θ* axis), but we also observe a collision avoidance response which depends on alignment: when the neighbour is in front and slightly on the left side of the focal individual (*θ*≃−*π*/6), this latter turns to the right, and its response is stronger if the neighbour is also oriented to the right, i.e. in a collision course with the focal individual. It is interesting to observe how the attraction and alignment responses are altered when we increase the temporal autocorrelation of noise. A longer temporal autocorrelation of noise means that if, for example, an individual is on the left of the trajectory that it is supposed to follow, it will also remain on the left of the trajectory for longer time before returning back to the target position. Under these conditions, the plots of Figure 4-(c) and (d) show a modulation along the alignment axis (*ϕ*). In fact, with correlated noise the particles retain their component of movement parallel to the common trajectory, while their attraction to the target position is comparatively weaker.

It is important to notice that the noise term in our simulations can be interpreted in two different non-exclusive ways. It can correspond to a real movement of animals constantly but imperfectly trying to keep a stable mutual position, but it can also correspond to tracking noise affecting the recorded trajectories of animals that do not move with respect to each other. To illustrate this, imagine the situation of two birds *i* and *j* sitting on a boat, such that they both move with respect to an external frame of reference, but the coordinates $\boldsymbol {X}_{\textit {ij}}^{real}(t)$ of bird *j* in the frame of reference of bird *i* are fixed $\boldsymbol {X}_{\textit {ij}}^{real}(t) = \boldsymbol {Const}$. Because of tracking noise, at any given time *t* we will record a relative position of the second individual with respect to the first $\boldsymbol {X}_{\textit {ij}}^{rec}(t) = \boldsymbol {X}_{\textit {ij}}^{real} + \boldsymbol {\eta }(t)$, where the recorded position $\boldsymbol {X}_{\textit {ij}}^{rec}(t)$ depends on the real position $\boldsymbol {X}_{\textit {ij}}^{real} $, and ***η***(*t*) is the displacement introduced by noise. If the noise is not correlated in time, the displacement ***η*** is expected to disappear at previous and subsequent instants of time: $\left \langle \boldsymbol {X}_{\textit {ij}}^{rec}(t-1)\right \rangle = \left \langle \boldsymbol {X}_{\textit {ij}}^{rec}(t+1)\right \rangle = \boldsymbol {X}_{\textit {ij}}^{real}$. On average over multiple observations, two animals whose recorded position and distance is $\boldsymbol {X}_{\textit {ij}}^{real} + \boldsymbol {\eta }(t)$ will revert to the real mutual position $\boldsymbol {X}_{\textit {ij}}^{real}$ and experience a movement −***η***(*t*) at the subsequent time interval. In such extreme case, the observed interaction responses between neighbouring individuals can completely be described by this ‘regression to the mean’ process, and the amplitude of ‘flocking responses’ is in direct proportion to the standard deviation of the noise. Temporal correlation in the noise retards this regression to the mean, and appears in the plots as an alignment response, because the autocorrelation preserves the component of movement parallel to the common trajectory of the pair, in spite of the fact that the two individuals are in their reciprocal attraction or repulsion zone, i. e. in spite that $\left |\boldsymbol {X}_{\textit {ij}}^{rec}(t)\right | \neq \left |\boldsymbol {X}_{\textit {ij}}^{real}\right |$.

**Figure 5:**
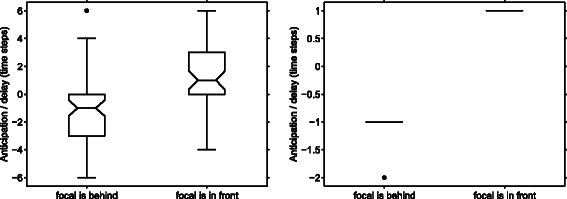
**Directional correlation delay vs. position in the group.** Each boxplot represents the distribution of directional correlation delays *τ* ^∗^ over simulated trajectories. The box on the left indicates trajectories in which the focal individual was in front; while the box on the right indicates those where the focal individual was behind. In our convention, positive values of the correlation delay *τ* ^∗^ indicate that the focal individual anticipates the changes of direction of its partner. When the individuals fly in a front-back configuration, measures of directional correlation indicate that the individual in front anticipates the turns of its neighbour. **Left** Individuals flying in a front-back configuration, temporal autocorrelation of the noise is short (*C*_*D*_=20 steps); 120 simulated trajectories. **Right** Same simulation parameters as for the figure on the left, but with longer temporal autocorrelation of noise (*C*_*D*_=100 steps). Note that in this case the variability is extremely reduced and *τ* ^∗^ was equal to ±1 in all but one simulation.

A number of recent studies have quantified leadership in collectively moving groups by computing directional correlation delays [14]. Directional correlation delays measure the characteristic delay within which one individual becomes aligned with a group neighbour, and it is assumed to indicate leadership behaviour if one individual consistently anticipates the direction taken by other members of the group. We computed directional correlation delays in our simulated data. When particles move side by side, there is no effect of being on the left or on the right, as we would have expected given the inherent left-right symmetry of the trajectories. When individuals move one behind the other, however, the individual in front appears to change direction first, and to be followed by its partner (see Figure 5). Intuitively we can see that when the common trajectory turns in one direction, the individual in front starts immediately turning in that direction, while the individual behind is projected temporarily to the opposite side of the curve. Increasing the temporal autocorrelation of noise does not change this, but it reduces the variability, because when errors on position are correlated, the estimation of direction of movement becomes more accurate.

**Figure 6:**
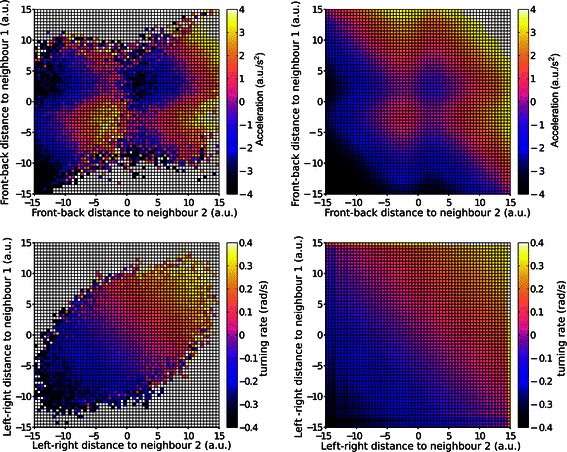
**Observed and predicted responses to multiple neighbours.** **Top row** Observed (left) and predicted (right) acceleration response in groups of three individuals. **Bottom row** Observed (left) and predicted (right) turning responses. Predicted responses are calculated by combining the observed responses in simulations with two individuals (one single neighbour) under the assumption that the combined effect of two neighbours is equal to the average of two independent pairwise responses. White squares in the grids on the left indicate missing values, never occurring in the simulations.

By generating trajectories with three or more individuals at a fixed distance from each other, we can test the apparent responses to multiple neighbours. Even if in our simulations the three individuals do not respond to each other, but simply try each to keep a constant distance and orientation relative to the common trajectory, this does not prevent us from studying how *apparent* responses to multiple neighbours are combined together. Figure 6 plots the observed turning (top row) and acceleration (bottom row) responses of a focal individual to two neighbours, for the case of three individuals moving in a front-back configuration. For this figure, the focal individual is randomly chosen between the three possible positions in the group (front, centre, back). The plots on the left in Figure 6 report the average responses of the focal individual as a function of the front-back distance of the first and second neighbour; the plots on the right report the turning and acceleration responses that would be predicted by averaging pairwise interactions, that is, if the response of the focal individual resulted from the average of two independent interactions with individual neighbours as those presented in the top row of Figure 3 (for comparison with a similar analysis on real fish interactions see Figure three of [10]). The combined responses to two neighbours are similar to those predicted from averaging pairwise interactions, but present larger modulations. This can be explained by considering that the position of all three individuals is affected by noise (or alternatively, that all three individuals can be randomly displaced by their target position). Hence, when the position of the focal individual appears to be displaced from its target relative to two neighbours, instead of just one, this provides increased evidence that the displacement is to be attributed to the focal individual, and not to the neighbours, and that the focal individual, and not one of the neighbours, is likely to show a compensatory response back to the target at the next time step.

## Discussion

In our analyses, the relative positioning of individuals, either side by side, or in a front-back configuration is sufficient to reproduce observed differences in the mechanisms used for collision avoidance, either by changing speed, or through turning. Anisotropic positioning of individuals with respect to their neighbours has been empirically observed in a number of species of collective moving animals, from fish [9-11,15] to birds [4,6,12] but it is not explicitly included into most self-propelled particle models of flocking and schooling. Some models involve a blind visual angle: a region of the visual field in which the presence of a neighbour does not induce any movement response (e.g. [16,17]), which can be considered as a form of anisotropy. However, these models otherwise consider attraction, alignment and repulsion as depending only on the distance from the neighbour, and not on its direction: interaction responses are organized in concentric regions around the focal individual. Outside animal behaviour, self-propelled particle models with anisotropic interaction zones have been studied in the context of collectively moving bacteria and other elongated or differently shaped particles (see e.g. [18,19]). In these systems, the repulsion zone is determined directly by steric occlusions, and it can lead to group formations organized in bands (smectic phases) [20]. In order to reproduce empirical observations, it seems important that future models of flocking and schooling take explicitly into account the anisotropy of interactions (it is bizarre how the empirical work of Ballerini and collaborators [4], one of the first detailed characterisations of anisotropic distribution of neighbours in flocks, triggered a large scientific debate about the topological - metric nature of interactions, but not about the anisotropy itself).

The interaction responses observed in our study can be interpreted in terms of animals constantly but imperfectly trying to keep an ideal mutual position. In theory, the same responses could also correspond to animals maintaining exactly the same ‘real’ positions relative to each other, but whose ‘recorded’ positions are affected by tracking noise. Because tracking noise induces similar apparent responses to real animal interactions, it is important that future studies try to achieve a precise understanding of the characteristics of the tracking noise, not only in terms of the amplitude of noise fluctuations, but also, and perhaps more importantly, of how these fluctuations are correlated in time. Temporal correlations in the noise can be introduced for instance by tracking algorithms that integrate prior expectations about the position of the target, which are relatively common features of GPS and video tracking software, and for this reason they are likely to be prevalent in empirical data sets. Our simulations show that these temporal correlations induce an apparent alignment response, because the autocorrelation preserves the component of movement parallel to the common direction of a group, in spite of the fact that the nearest neighbours are in their reciprocal attraction or repulsion zones.

The flocking interactions observed in our study represent responses around a fixed point. They describe the continuous adjustments that allow a flock or school to maintain a preferred configuration as the group moves. As such, they are not necessarily informative about when and how navigational decisions are taken: we would observe them even in the extreme case in which individuals have perfect agreement about the route to follow. Our simulations do actually imply such an agreement about a common route, in the sense that both particles follow the common trajectory with similar responses and no conflict. We can speculate that precisely in the presence of navigational conflict, the equilibrium of mutual arrangements will be destabilized: interactions with environmental stimuli interfere with neighbour to neighbour interactions and induce individuals to abandon their mutual relative positions and alignment. This is in part captured by common measures of movement leadership such as the directional correlation delay [14], which implicitly assumes that leaders are those individuals that abandon more often their orientation parallel to the neighbour, and followers are those individuals with a higher tendency to restore the aligned group configuration. In our analyses, directional correlation delays correlate with the position in front or on the back of the group. If we do not assume that trajectories are pre-imposed, but result from interactions, the individual that moves in front is also the first to draw the common trajectory, and it is reasonable to impute route decisions to this individual.

One of the open problems in research on collective motion is that of determining how individuals combine interactions with multiple neighbours. Here, we have shown that multiple neighbours can carry additional information about the movement of a focal individual not directly because they take part in the interactions, but indirectly because they reduce our uncertainty about the real position of the focal individual. If an animal group maintains a ‘solid-like’ configuration, whereby individuals keep a constant position relative to their neighbours most of the time, like in our trajectories, the movement of a focal individual can be predicted in terms of its response to a single nearest neighbour, and including information about additional neighbours reduces uncertainty, but apart from this does not bring additional information. This might explain why information theoretical approaches, like the one adopted in [11] indicated that the movement of a focal individual can be predicted to a large amount by looking at only one nearest neighbour, and including further neighbours only marginally helped to improve the prediction. It had already been noted [21] that interaction responses cannot be correctly inferred if interactions only take place close to steady-state positions, as opposed to transient non steady state positions. We are confident that future studies discriminating between interactions around a stable mutual position and transient interactions in which the mutual positions are abandoned will help to further improve our understanding of more complex patterns of response to multiple neighbours.

## Conclusion

We have illustrated the duality between interaction rules and mutual positions in moving animal groups. The duality can be stated as follows: (1) if the interactions among neighbours are anisotropic, this leads to consistent patterns of positioning of an animal relative to its neighbours, and (2) if animals aim at keeping a particular position relative to their neighbours, this can only be achieved through interaction responses with specific anisotropic characteristics.

Our analyses suggest that movement interactions observed and quantified by recent studies on real animal group are largely determined by simple positional adjustments necessary to maintain a preferred local configuration of the group, and point to the necessity of discriminating between these interactions around a stable mutual position, and interactions that correspond to real navigational decisions.

Because tracking noise has analogous effects to interactions around a stable mutual position, it is important that future empirical studies take explicitly into account the effects of noise based on its amplitude and its temporal correlation patterns.

## Methods

### Trajectory generation

We generated random trajectories, each having a length *N*=2^12^ steps. The trajectories are defined by a sequence of step lengths (speed per time step) and a sequence of turnings intercalated between the steps.

The speed values *S* are numbers extracted from the distribution

(1) $$ S = S_{0} + s \frac{\epsilon_{1}(t)}{\max \left| \epsilon_{1} \right|}  $$

and the turning angles *T* are

(2) $$ T = a \frac{\epsilon_{2}(t)}{\max \left| \epsilon_{2} \right|}  $$

In these equations, *ε*_1_ and *ε*_2_ represent sequences of temporally correlated random numbers and are generated as follows. We first generate *N* random numbers uniformly distributed in the interval [−0.5,0.5]. In order to exclude abrupt changes of direction and speed, we apply to both sequences a low-pass temporal frequency filter with equation

(3) $$ \epsilon(t) = \exp \left(- \frac{\omega(t)^{2}}{2\sigma^{2}} \right)   $$

where *ω* are temporal frequencies and *σ* controls the filter standard deviation. By setting $\sigma = \frac {N}{C_{T}}$, with a cut-off period for the temporal correlations *C*_*T*_=300 steps we impose that speed and turning fluctuations typically occur over a period of 300 time steps, or longer. In our simulations, we fix arbitrarily *S*_0_=5 and *s*=0.2 arbitrary units (a.u.) per time step and *a*=0.02 radians per time step. We further assume that 5 time steps in the trajectory correspond to one second of time. Our results are intended to illustrate qualitative differences in the observed patterns of movement, which remain stable for wide ranges of arbitrary parameters.

The positions of individuals along the trajectory at time *t* are determined by first drawing the segment that intersects the trajectory at *t* and having a specific orientation *θ* relative to the segment of trajectory between *t* and *t*+1, and selecting equally spaced points (at distance *r*=5 a.u. from each other) on this segment. These individual trajectories represent the movement of an hypothetical focal individual and its partner (and in some simulations of a third individual) which successfully keep a constant distance and relative position to each other while moving together.

The ‘recorded’ positions of the individuals do not match exactly those generated as above, but are displaced in a random direction at every time step, to simulate tracking noise, or an imperfect ability to maintain the desired flocking configuration. These displacements are autocorrelated in time, so that if an individual is for instance on the left of its target position at time *t*, it is more likely to be on the left of the target position also at time *t*+1. There is no cross-correlation between the random displacements of the focal individual and those of its neighbour. The random displacements are computed as follows. We first generate series of *N* random numbers, normally distributed with mean 0 and standard deviation 1, then we apply a low-pass filter analogous to the one used in Equation 3, with cut-off frequency $\sigma _{d} = \frac {N}{C_{D}}$, where *C*_*D*_ is the cut-off correlation period for displacements (the number of time steps after which the displacements become uncorrelated). In our simulations *C*_*D*_=20 steps except when otherwise stated. After the filtering operation, we rescale the numbers to obtain distributions with standard deviation *r*/2. Two random numbers taken from two such generated series describe the x and y components of the displacement.

The analyses reported in the present manuscript focus on the comparison of two conditions. In the first condition the focal individual has a target position directly in front or behind its neighbour (*θ*=0). In the second condition, the target position for the focal individual is on the side of its neighbour (*θ*=*π*/2). For each condition, we generate 100 random trajectories. The order of individuals along the segment, that is, whether the focal individual is in front or behind its neighbour (respectively left or right when *θ*=*π*/2) is constant for the whole length of one trajectory, but changes randomly from one trajectory to the other, with half of the trajectories on average displaying the focal individual on the left and the other half displaying it on the right. The movement responses observed in all trajectories are merged together for the analyses.

### Data analysis

At each time step *t* we measure the instantaneous speed of the focal individual

$$s(t) = \sqrt{\left(x(t) -x(t-1) \right)^{2} + \left(y(t) -y(t-1)\right)^{2}}/dt, $$

where *x*(*t*) and *y*(*t*) are the *x* and *y* coordinates of the focal individual at time *t* and *dt* is the duration of a time step. The direction of movement of the focal individual is

$$\psi(t) = \mathrm{atan2}\left(y(t)- y(t-1), x(t)- x(t-1)\right),. $$

The response of the focal individual to its neighbours is described by its tangential acceleration

$$a(t) = \left(s(t) - s(t-1)\right) / dt $$

and its speed of direction change

$$\alpha(t+1) = \left(\psi(t) - \psi(t-1)\right)/dt, $$

where care is taken to compute the correct angular difference, *ψ*(*t*)−*ψ*(*t*−1), with regard to the periodicity of *ψ*(*t*).

The relative position and orientation of a neighbour in the frame of reference of the focal individual are described by their observed mutual distance

$$d_{ij} \left(t \right) = \sqrt{\left(x_{j}(t) -x_{i}(t) \right)^{2} + \left(y_{j}(t) -y_{i}(t)\right)^{2}}, $$

and the direction *θ* of the neighbour in the frame of reference of the focal fish was

$$\vartheta_{ij}(t) = \mathrm{atan2} \left(y_{j}(t) - y_{i}(t), x_{j}(t)- x_{i}(t) \right) - \alpha_{i} \left(t \right). $$

The directional correlation delay *τ*^∗^ is the time delay *τ* that maximizes the correlation of direction between the focal individual and its partner

$$ \tau_{ij}^{*} = {\underset{\tau}{\text{arg~max}}} \left\langle \cos \left(\psi_{i}(t) - \psi_{j}(t + \tau) \right) \right\rangle $$

The Matlab®; source code used to generate the trajectories and for all the analyses is available as online Additional file 1.

## Additional file

Additional file 1
**Compressed folder containing all the Matlab®; script files required to repeat the analyses reported in this paper.**
